# The neural substrates of social cognition deficits in newly diagnosed multiple sclerosis patients

**DOI:** 10.1002/acn3.52085

**Published:** 2024-06-13

**Authors:** Stefano Ziccardi, Helen Genova, Elisa Colato, Maddalena Guandalini, Agnese Tamanti, Massimiliano Calabrese

**Affiliations:** ^1^ Neurology Section, Department of Neurosciences, Biomedicine and Movement Sciences University of Verona Verona Italy; ^2^ Kessler Foundation 120 Eagle'Rock Ave, Suite 100 East Hanover New Jersey 07936 USA; ^3^ Department of Physical Medicine and Rehabilitation, New Jersey Medical School Rutgers University Newark New Jersey 07101 USA; ^4^ MS Centre, Department of Anatomy and Neuroscience Amsterdam UMC Amsterdam the Netherlands

## Abstract

**Objective:**

Cognitive and affective symptoms in multiple sclerosis (MS) can be independently impaired and have different pathways of progression. Cognitive alterations have been described since the earliest MS stages; by contrast, the social cognition (SC) domain has never been investigated in the first year from MS diagnosis. We aimed to evaluate SC and unravel its neural bases in newly diagnosed MS patients.

**Methods:**

Seventy MS patients underwent at diagnosis a 3 T‐MRI and a neuropsychological/SC assessment (median time between diagnosis and MRI/cognitive evaluation = 0 months). We tested two matched reference samples: 31 relapsing–remitting MS patients with longer course (mean ± SD disease duration = 7.0 ± 4.5 years) and 38 healthy controls (HCs). Cortical thicknesses (CTh) and volumes of brain regions were calculated.

**Results:**

Newly diagnosed MS patients performed significantly lower than HCs in facial emotion recognition (global: *p* < 0.001; happiness: *p* = 0.041, anger: *p* = 0.007; fear: *p* < 0.001; disgust: *p* = 0.004) and theory of mind (*p* = 0.005), while no difference was found between newly diagnosed and longer MS patients. Compared to lower performers, higher performers in facial emotion recognition showed greater volume of amygdala (*p* = 0.032) and caudate (*p* = 0.036); higher performers in theory of mind showed greater CTh in lingual gyrus (*p* = 0.006), cuneus (*p* = 0.024), isthmus cingulate (*p* = 0.038), greater volumes of putamen (*p* = 0.016), pallidum (*p* = 0.029), and amygdala (*p* = 0.032); patients with higher empathy showed lower cuneus CTh (*p* = 0.042) and putamen volume (*p* = 0.007).

**Interpretations:**

SC deficits are present in MS patients since the time of diagnosis and remain persistent along the disease course. Specific basal, limbic, and occipital areas play a significant role in the pathogenesis of these alterations.

## Introduction

Multiple sclerosis (MS) is a chronic disease of the central nervous system characterized by sequelae of inflammation and neurodegeneration.^1^ MS is the most common nontraumatic neurological cause of disability in young adults, resulting in a dramatic impact on the quality of life of patients and their caregivers.^2^ In addition to physical symptoms, patients with MS exhibit a wide range of “invisible symptoms” during the disease course: among these, cognitive and emotional difficulties are frequently reported.^3^

Cognitive alterations in MS show a prevalence of up to 65% depending on classification methods and neuropsychological batteries/tests used.^4^ Cognitive alterations have been described also in patients at the earliest stages of the disease and in those who are not characterized by a clinically defined cognitive impairment,^5^ with a dramatic burden on familiar, vocational, and social spheres of patients.^6^ Traditionally, cognitive domains mainly affected in MS are information processing speed, attention, memory, and executive functioning.^7^

Nevertheless, evidence has been proposed on a new domain in which MS patients show specific deficits: social cognition (SC). Interest in the concept of SC has grown over the last 20 years. In 2013, SC was included in the last revision of the Diagnostic and Statistical Manual of mental disorders (DSM‐V) as one of the six key neurocognitive domains. SC refers to the information about people and social situations; it includes all the key mental operations underlying social interactions and processes required to establish and sustain interpersonal relationships.^8^, ^9^ SC is a multidimensional domain that includes facial emotion recognition, theory of mind, and empathy.^10^ Facial emotion recognition is the process of identifying human emotions from facial expressions.^11^ Theory of mind (ToM) is defined as the ability to infer the intentions, dispositions, and beliefs of others.^12^ Empathy refers to the capacity to feel and understand what another person is experiencing.^13^ SC is a complex construct involving wide neural networks, resulting in deficits in different neurological conditions (e.g., Alzheimer's disease, Parkinson's disease, Huntington's disease, and frontotemporal dementia).^14^, ^15^, ^16^, ^17^

Cognitive impairment has been well described in MS; however, assessment of SC deficits is still not part of MS routine clinical practice, despite evidence that they are frequent in MS. Previous studies demonstrated that MS patients showed a significantly lower performance, compared to healthy controls, in all subdomains of SC,^18^, ^19^, ^20^ affecting patients independently from MS subtype, physical disability, and disease duration,^8^, ^21^, ^22^, ^23^ and that this impairment tends to be consistent over time.^24^ It is also important to underline that, despite associations have been described between traditional cognitive domains and SC,^8^, ^25^, ^26^, ^27^, ^28^, ^29^, ^30^, ^31^, ^32^ MS patients showed SC deficits even in the absence of traditional cognitive impairment, suggesting the potential independence of this domain.^19^, ^22^, ^24^, ^33^

These alterations have been linked to specific neural bases. Previous studies demonstrated the involvement of the amygdala as the key region of social cognition functioning, showing an association between SC performance and amygdala atrophy^34^ and lesion volume.^19^, ^24^ Moreover, also the insular and medial frontal cortices have been identified as important structures for SC tasks.^35^ In addition, some studies have been conducted investigating neural correlates of SC using functional MRI: alterations in resting state functional connectivity between specific brain regions, such as amygdala, fusiform gyrus, and lateral occipital cortex,^33^, ^36^ as well as specific networks (i.e., default mode network, executive network, and limbic‐paralimbic network), have been described.^37^, ^38^

As mentioned above, impairment in SC can lead to incorrect identifications of emotions, intentions, and thoughts in social situations, resulting in inappropriate social interactions, causing interpersonal conflicts and negative effects on interpersonal relationships, work status, and familiar quality of life.^10^, ^24^ Thus, it is crucial to identify SC deficits since the early stages of MS. Even though SC deficits have been described in patients with MS, it is still partially unknown the timing in which these alterations emerge, and therefore if also early MS patients are characterized by some sort of SC impairment. Most of the studies evaluated SC in MS were conducted with heterogeneous cohorts of patients with a long disease course and a severe physical/cognitive burden. To the best of our knowledge, only three studies have been so far conducted evaluating SC functioning in early MS patients. The first study evaluated SC in 25 relapsing–remitting MS (RRMS) patients within 2 years from diagnosis: Authors reported that RRMS showed worse performance on ToM task, compared to HCs, and perceived themselves as less empathetic.^28^ A more recent study, in which 34 RRMS underwent SC tests within 2 years from diagnosis, demonstrated worse performance in a task of ToM and a task of facial emotion recognition, while no difference has been found for empathy.^39^ Lastly, a new study examined 38 RRMS patients within 5 years from diagnosis and showed a specific alteration in ToM, compared to HCs, but not in the domain of facial emotion recognition.^40^ Therefore, these studies concluded supporting the evidence of the presence of SC deficits since the early stages of MS that might negatively influence interpersonal relationships.

Despite being of great relevance, several questions remain, which we will attempt to address in the current study: are SC alterations already present at the time of MS diagnosis, at the very beginning of the disease? And, since previous studies did not include MRI data, which are the neural bases of these early deficits? We aimed to conduct an early comprehensive assessment of cognitive/SC functioning. Further, we investigated MRI outcomes in a homogeneous sample of MS patients at the time of diagnosis, comparing their behavioral performance to a group of MS patients with longer disease duration and to a matched group of healthy controls. We hypothesized that regional structural damage found associated with SC performance in MS patients after a consistent time from the diagnosis would be also related to SC deficits since the earliest stages of the disease.

## Methods

### Study design and participants

In the present study, we enrolled 70 MS patients at the time of their diagnosis, and all recruited at the MS Centre of the Veneto Region (Verona, Italy).

We also included two reference samples: 31 relapsing–remitting MS (RRMS) patients during their MS course (mean ± SD disease duration = 7.0 ± 4.5 years) and 38 healthy controls (HCs). All the three groups were matched for age, sex, and education.

All MS patients underwent neurological examinations investigating physical disability using the Expanded Disability Status Scale (EDSS).^41^

Exclusion criteria were the presence of any concomitant neurological condition (other than MS), any psychiatric or other pathological conditions, substance abuse, hearing impairment, and any upper limbs. Primary‐progressive patients have not been included.

The study was conducted in accordance with the Declaration of Helsinki (2013) and was approved by the local Ethics Committee of Verona (1621‐CESC). Informed consent was obtained.

### Neuropsychological assessment

All MS patients underwent a comprehensive neuropsychological assessment at diagnosis (median time between diagnosis and neuropsychological assessment = 0 months, IQR = 3.0, range = 0–12) and in a clinically stable phase (no relapses/acute corticosteroid treatments in the 30 days before) by using the Brief Repeatable Battery of Neuropsychological Tests (BRB‐NT)^42^ and the Stroop Test.^43^ The BRB‐NT is composed of tests of verbal learning and delayed memory recall (Selective Reminding Test, SRT), visuospatial learning and delayed memory recall (10/36 Spatial Recall Test, SPART), visual information processing speed and attention (Symbol Digit Modalities Test, SDMT), auditory information processing speed, attention, and calculation (Paced Auditory Serial Addition Task, PASAT), and semantic verbal fluency (Word List Generation, WLG). The ST is a test of attention and automatic response inhibition (ST‐EIT and ST‐EIE).

Raw scores were corrected by adjusting for age, education, and sex according to the Italian normative data of each test. Adjusted scores below the cutoff (5th percentile) were classified as failed. MS patients were classified into three groups based on their performance on all the neuropsychological tests administered, using a conservative approach^44^: “cognitively normal” (CN, 0 failed subtests), and “cognitively impaired” (CI), specifically “mildly cognitive impaired” (mCI, up to 2 failed subtests) or “severely cognitive impaired” (sCI, at least three failed subtests).

To control for the emotional state, the Depression Anxiety Stress Scale 21‐item (DASS‐21)^45^ was administered.

### Social cognition protocol

We investigated the three subcomponents of the SC domain (facial emotion recognition, theory of mind, and empathy) in all MS patients with the same protocol used and described in previous studies.^19^, ^24^

Facial emotion recognition was evaluated by means of the Task of Facial Emotion Recognition‐Kessler Foundation (TOFER‐KF).^46^ The TOFER‐KF is composed of 36 black‐and‐white photographs of human faces displaying one of the six basic emotions (happiness, anger, fear, sadness, surprise, and disgust). The stimuli were taken from the well validated Karolinska Directed Emotional Faces (KDEF) database.^47^, ^48^ Each emotion was randomly presented on a screen six times (6 emotions each for 6 times = 36 stimuli in total). Participants were asked to choose which emotion each face was expressing. As a control task, participants were also asked to indicate the gender of each face. For each correct response, one point was assigned, while no points were assigned for incorrect responses (maximum score = 36).

Theory of mind (ToM) was evaluated using the Italian version of the Reading the Mind in the Eyes test (RME).^49^ The RME is composed of 36 black‐and‐white images representing the eyes of different individuals. Each image was shown on a screen for 5 seconds; subsequently, on each corner of the screen, four emotional adjectives were presented. Participants were asked to choose which of the four adjectives best described the emotion transmitted by the eyes. As a control task, participants were also asked to indicate the gender of each face. The four adjectives in each image shared the same valence (e.g., “terrified,” “upset,” “arrogant,” and “annoyed”). For each correct response, one point was assigned, while no points were assigned for incorrect responses (maximum score = 36).

The Empathy Quotient (EQ)^50^ is a self‐reported questionnaire assessing the level of empathy. The EQ is composed of 60 items (40 about empathy and 20 about other domains), each with a 4‐point Likert‐scale answer. For the 40 empathy questions, 21 were empathetic statements (0 points: “strongly disagree” and “slightly disagree,” 1 point: “slightly agree,” and 2 points: “strongly agree”), while 19 were nonempathetic statements (0 points: “strongly agree” and “slightly agree,” 1 point: “slightly disagree,” 2 points: “strongly disagree”). No points were assigned to the 20 nonempathic questions that served as control tasks. The maximum total score is 80: the higher the total score, the higher the level of empathy.

### MRI acquisition

The experimental group underwent a 3.0 T MRI (Phillips Medical Systems, Best, The Netherlands) at diagnosis close to the neuropsychological and social assessment (median time between clinical assessment and MRI = 0 months, IQR = 1.0, range = 0–6).

The following sets of images were acquired:

- 3D fluid attenuated inversion recovery (FLAIR) repetition time (TR)/echo time (TE) = 5500/292 ms, inversion time (TI) = 1650 ms, voxel dimension of 1 × 1 × 1 mm^3^, matrix = 256 × 256;
- 3D double inversion recovery (DIR) TR/TE = 5500/292 ms, TI1/TI2 = 525/2530 ms voxel dimension of 1 × 1 × 1 mm^3^;
- 3D T1‐weighted fast field echo (FFE) TR/TE = 8.4/3.7 ms, voxel dimension of 1 × 1 × 1 mm^3^, matrix = 256 × 256;
- 2D T1w spin echo Gadolinium (SE GD): TR/TE = 550/10 ms, 50 contiguous axial slices with a thickness = 3.0 mm, matrix = 256 × 256.

### MRI imaging analysis

Cortical thicknesses and volumes of specific brain regions were calculated for each of the volumetric T1‐weighted data set using FreeSurfer,^51^ available online (http://surfer.nmr.mgh.harvard.edu). Volumes of subcortical structures (thalamus, caudate, putamen, pallidum, hippocampus, amygdala, and nucleus accumbens) were calculated using the FIRST tool.^52^

### Statistical analysis

Statistical analyses were performed using SPSS statistic software (SPSS Inc., Chicago, IL, USA, version 24). The normality distribution of data was checked by using the Shapiro–Wilk test.

To evaluate SC functioning at the time of MS diagnosis, we compared scores between patients at diagnosis and both other MS patients and HCs. We also evaluated SC at diagnosis in associations with other potential confounders, comparing scores among patients with different levels of global cognitive impairment, with different MS diagnoses, and Pearson or Spearman correlation (depending on normality distribution) was conducted to investigate the association with emotional state.

Moreover, for each social cognition task, we divided the sample into lower performers (scores below the average of the sample) and higher performers (scores above the average of the sample). We used *t*‐tests or Mann–Whitney tests (depending on normality distribution) to compare MRI data between lower and higher performers in each SC test.

Results were presented as mean ± SD for continuous variables or as median [interquartile range (IQR)] for discrete variables. Effect sizes are reported in terms of Cohen's d. A p‐value less than 0.05 was considered statistically significant.

## Results

### Clinical and demographic characteristics of subjects

The experimental group was composed of MS patients at the time of diagnosis: 49 females (70.0%), mean ± SD age = 38.0 ± 11.7, mean ± SD education = 14.6 ± 3.1. According to the most recent MS diagnostic criteria,^53^ the experimental sample was composed of 56 (80.0%) RRMS and 14 (20.0%) clinically isolated syndromes (CIS). Median EDSS was 1.5 [1.25] (Table 1).

**Table 1:** Clinical and demographic characteristics of the samples.

	MS patients at diagnosis (*n* = 70)	RRMS patients with a longer MS course (*n* = 31)	Healthy controls (*n* = 38)	MS patients at diagnosis vs RRMS with a longer MS course	MS patients at diagnosis vs healthy controls
Gender (M/F)	21/49	7/24	10/28	n.s.	n.s.
Age (years)	38.0 ± 11.7	36.3 ± 7.6	37.1 ± 8.9	n.s.	n.s.
Education (years)	14.6 ± 3.1	13.4 ± 3.4	14.6 ± 3.4	n.s.	n.s.
Clinical course (CIS/RR)	14/56	0/31	–	–	–
Disease duration from diagnosis (years)	0.2 ± 0.3	7.0 ± 4.5	–	*p* < 0.001	–
EDSS	1.5 [1.25]	1.0 [3.5]	–	*p* = 0.002	–

Mean ± SD was reported for continuous variables, while median [IQR] was reported for discrete variables.

n.s., not significant.

### Behavioral results

Forty‐seven patients (67.1%) were CN, 15 patients (21.4%) were mCI, and 8 patients (11.4%) were sCI. No significant differences were found in all SC domains between patients at diagnosis with different levels of cognitive impairment.

At diagnosis, RRMS patients at diagnosis showed significantly lower self‐reported empathy compared to CIS patients (*p* = 0.016), while facial emotion recognition and ToM did not show significant differences.

No significant correlations were found between SC global scores and emotional state (DASS‐21) at diagnosis, both considering the global score and also the single subscales of depression, anxiety, and stress.

Compared to HCs, patients with MS at the time of diagnosis showed significantly lower scores in facial emotion recognition, both at a global level (*p* < 0.001) and regarding specific emotions (happiness: *p* = 0.041, anger: *p* = 0.007, fear: *p* < 0.001, disgust: *p* = 0.004). Significantly lower scores were also found in patients with MS at diagnosis, compared to HCs, in the RME task (*p* = 0.005). No difference was found in the empathy questionnaire.

Interestingly, when the social cognition scores were compared between MS patients at diagnosis and those with a longer disease course, no differences were found in all three global performances, and also considering facial emotion recognition of single emotions.

**Figure 1:**
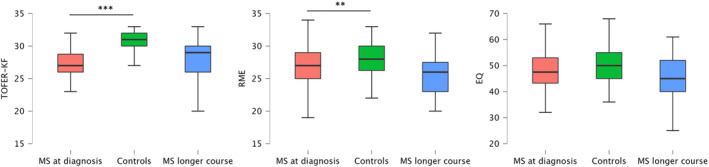
Global performance in the three social cognition domains among the three groups evaluated: the experimental groups (MS patients at diagnosis, red‐colored) and the two reference groups (healthy controls, green‐colored, and MS patients with a longer course of disease, blue‐colored). Highlighted the results of the statistical comparisons between the experimental group and the two control groups. **p < 0.01; ***p < 0.001.

Detailed results are shown in Figure 1.

### MRI results

In the experimental group, the mean ± SD global score for facial emotion recognition was 27.2 ± 2.5: 38 patients at diagnosis were lower performers (mean ± SD = 25.2 ± 2.0) and 32 were higher performers (mean ± SD = 29.6 ± 1.6). The mean ± SD score for ToM was 26.4 ± 3.4: 33 patients at diagnosis were lower performers (mean ± SD = 23.8 ± 2.7) and 37 were higher performers (mean ± SD = 28.8 ± 1.8). The mean ± SD score for EQ was 48.0 ± 8.6: 34 patients at diagnosis were lower performers (mean ± SD = 41.5 ± 6.1) and 36 were higher performers (mean ± SD = 54.6 ± 5.1).

**Figure 2:**
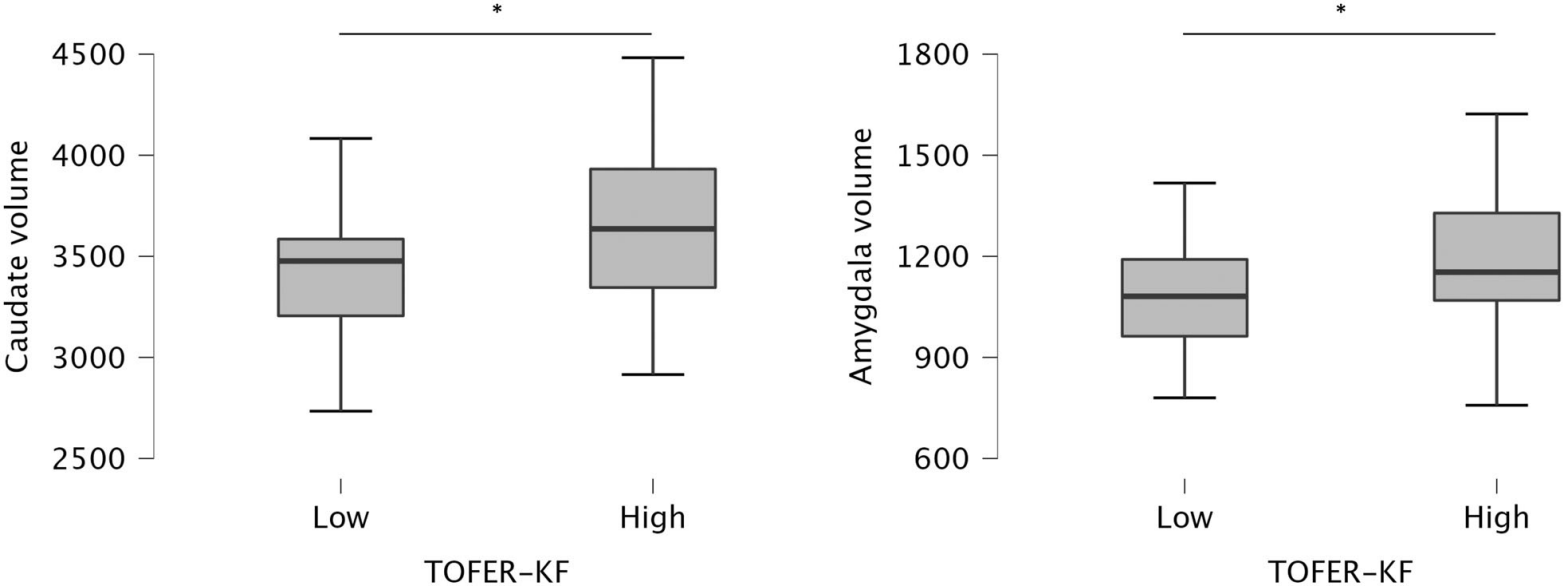
Significant differences in terms of MRI results between MS patients lower and higher performers in the facial emotion recognition task. *p < 0.05.

Considering global facial emotion recognition, a statistically significant difference emerged for the volume of amygdala (*p* = 0.032, *d* = 0.5) and caudate (*p* = 0.036, *d* = 0.5): at the time of diagnosis, higher performers showed a greater volume of amygdala and caudate compared to lower performers (Fig. 2).

**Figure 3:**
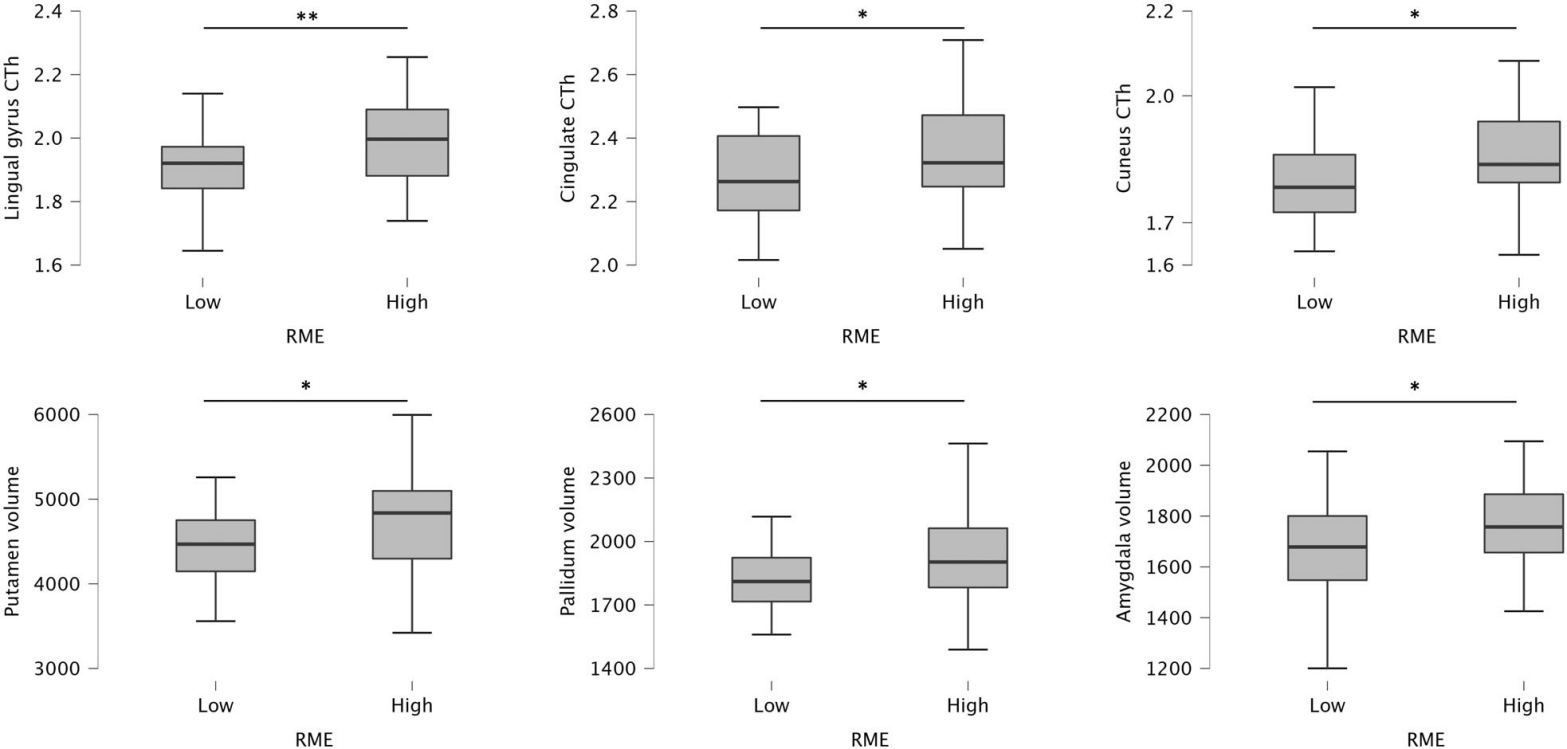
Significant differences in terms of MRI results between MS patients lower and higher performers in the theory of mind task. *p < 0.05; **p < 0.01.

Considering ToM, a statistically significant difference emerged for the cortical thicknesses of lingual gyrus (*p* = 0.006, *d* = 0.7), cuneus (*p* = 0.024, *d* = 0.6), and isthmus cingulate (*p* = 0.038, *d* = 0.5), and for the volumes of putamen (*p* = 0.016, *d* = 0.5), pallidum (*p* = 0.029, *d* = 0.4), and amygdala (*p* = 0.032, *d* = 0.5): at the time of diagnosis, higher performers showed a greater cortical thickness and volume in these regions/structures compared to lower performers (Fig. 3).

**Figure 4:**
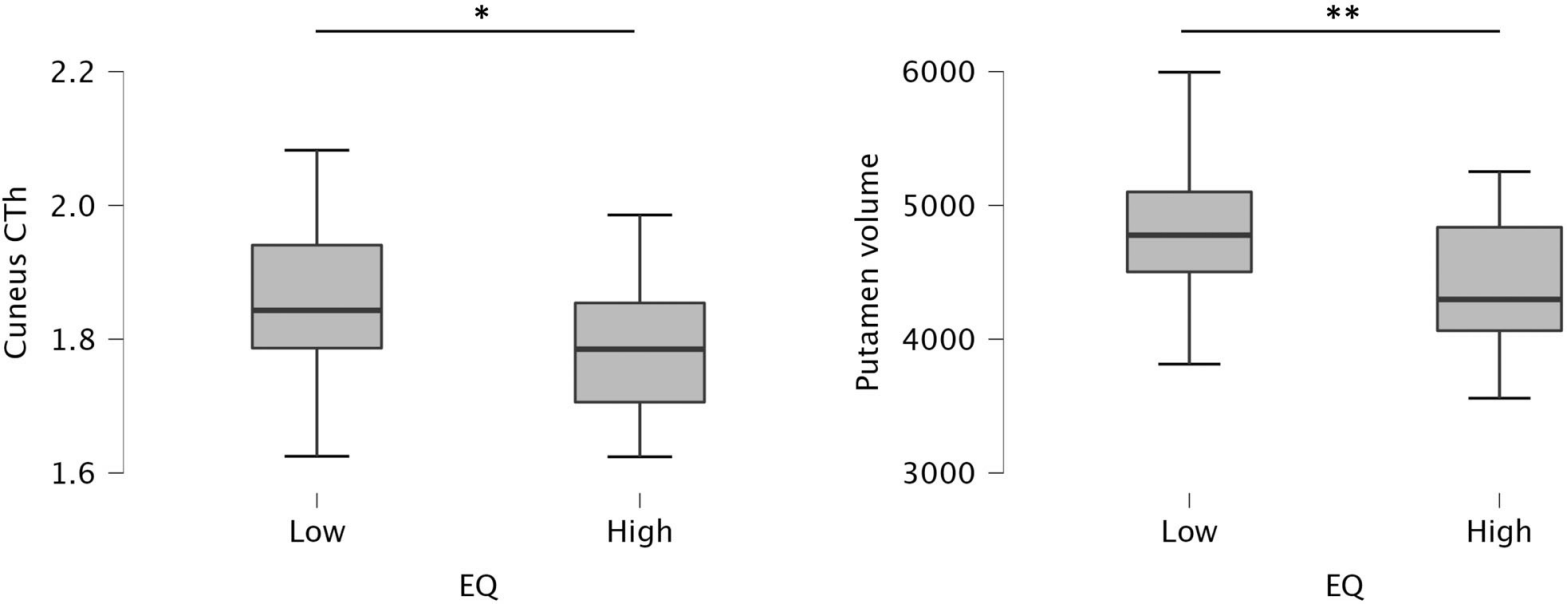
Significant differences in terms of MRI results between MS patients lower and higher performers in the empathy questionnaire. *p < 0.05; **p < 0.01.

Considering empathy, a statistically significant difference emerged for the cortical thickness of cuneus (*p* = 0.042, *d* = 0.5), and the volume of putamen (*p* = 0.007, *d* = 0.6): higher performers showed a greater cortical thickness and volume in these regions/structures compared to lower performers (Fig. 4).

**Figure 5:**
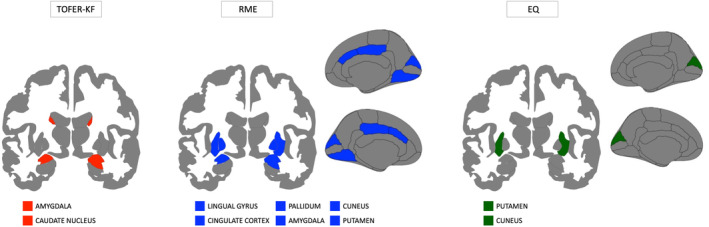
Brain representations showing regions of interest that showed a significant association with the three domains of social cognition: facial emotion recognition (TOFER‐KF test, red‐colored); theory of mind (RME test, blue‐colored); empathy (EQ questionnaire, green‐colored).

Significant regions associated with specific SC components have been graphically reported in Figure 5.

## Discussion

Social cognition alterations have been reported in patients with MS, as well as the negative impact resulting on the quality of life of patients and caregivers. However, social abilities are often neglected, and their assessment is not included in the MS routine clinical practice.

Our study highlights the presence of social cognition deficits already at the time of diagnosis in MS patients. Newly diagnosed MS patients showed a significantly lower performance compared to a group of healthy controls in tests of facial emotion recognition and of theory of mind, two key aspects of the social domain. In particular, facial recognition of specific emotions was impaired, such as happiness, anger, fear, and disgust, replicating previous literature.^19^ Moreover, we also demonstrated that, when compared to a group of MS patients with a longer disease course, newly diagnosed MS patients showed a comparable SC performance in the two abovementioned abilities and also in self‐reported empathy. These results suggest that, already at diagnosis, MS patients are characterized by alterations in SC functioning, which however tend to remain stable along the disease course. This is in line with our previous longitudinal study that demonstrated the permanence of SC deficits in a group of MS patients after 3 years of follow‐up.^24^ Considering the trend of SC performances over time in our three groups (controls, MS patients at diagnosis, MS patients with a longer disease course), SC alterations might reflect a prodromal sign for MS that can be helpful even in the prediagnostic phase, as previously demonstrated in other neurodegenerative and psychiatric conditions such as Alzheimer's disease^54^ and psychosis.^55^, ^56^, ^57^ Moreover, it is noteworthy that SC alterations were independent from both cognitive impairment and psychological status, since no association was found at diagnosis with global cognitive status and a depression/anxiety/stress scale.

Few previous studies conducted on recently diagnosed MS patients reported difficulties in SC performance.^28^, ^39^, ^40^ Nevertheless, the present study consisted of a larger sample than what has been examined previously (70 vs less than 40). Moreover, in addition to comparing to healthy controls, we have included another group of MS patients with a longer disease course. Thus, comparing our sample to two different reference groups, we have characterized the phenomenon of social cognition deficits at different clinical courses (either at diagnosis or not). Furthermore, we included an MS sample, who underwent the neuropsychological assessment and the SC protocol in close proximity to their diagnosis, with a mean time between diagnosis and cognitive evaluation was equal to 0 (other studies considered MS patients within 2 or 5 years from diagnosis).

Our study also examined the neural bases of SC impairments using MRI outcomes, which has not been done previously in newly diagnosed participants. Cortical atrophy and subcortical regional volume loss across the brain due to MS are not uniform,^58^, ^59^, ^60^, ^61^ since some areas are more susceptible than others to neurodegeneration.^62^, ^63^ For this reason, instead of considering global MRI measures, it has been suggested to focus on brain regional volumetric outcomes.^64^ Results showed that SC deficits at the time of diagnosis rely on specific regional neural bases, with medium/large effect sizes (Cohen's d between 0.4 and 0.7). Specifically, facial emotional recognition was associated with basal ganglia and limbic system: higher performers showed a significantly higher volume of amygdala and caudate compared to lower performers. The amygdala has been widely recognized as probably the key brain region for discriminating facial emotions,^10^, ^34^, ^65^, ^66^ and its role in MS patients has been demonstrated in two previous publications from our group,^19^, ^24^ while also caudate association with SC domain has been recently reported.^67^ Moreover, ToM performance was related to cortical thickness in the lingual gyrus, the cingulate, and the cuneus, as well as volume in the putamen, the pallidum, and the amygdala: as for facial emotion recognition, higher performers showed a significantly greater cortical thickness and volume in these regions compared to lower performers. Cingulate is one of the brain areas most related to social abilities,^35^, ^65^, ^66^ in particular involved in ToM,^67^, ^68^ as well as other cognitive deficits in MS patients,^58^, ^69^, ^70^, ^71^, ^72^, ^73^ probably since it is one of the first regions becoming atrophic in relapsing–remitting MS patients.^64^ In addition to the abovementioned amygdala, the pallidum appeared to be a subcortical structure influencing ToM performance, in agreement with a previous study evaluating MRI correlates of ToM abilities.^68^ Lastly, empathy was also associated with MRI outcomes: more empathetic MS patients showed lower cuneus thickness and lower putamen volume. Even seeming counterintuitive, this result has to be considered with caution since empathy is measured through the administration of a self‐reported questionnaire; nevertheless, it appears to agree with previous studies showing higher empathy levels to be related to reduced gray matter volume.^74^

We are aware that the present study is not free from limitations. First of all, separating the MS sample into lower and higher performers offers an interesting approach to consider data, especially for a diagnostic approach, however, it may not have given us enough power to truly examine the research questions. Future studies should consider increasing the sample size. In addition, the common delay that MS patients experience to get the diagnosis might have influence the disease duration and the interpretation of SC deficits; however, our two groups of patients (either at diagnosis or not) showed the same limited diagnostic delay. Moreover, additional MRI data would have helped to understand pathological substrates of SC difficulties: future studies could consider evaluating patients with advanced MRI sequences, such as functional MRI, or by evaluating also focal measures of damage (e.g., white matter and cortical lesions, described as two main pathological factors of MS that could lead to cognitive difficulties). Furthermore, it is difficult to fully interpret our results because of the potential influence of disease modifying therapies which may have stabilized progression of social cognition. Lastly, future research could benefit from using more ecological and dynamic measures to assess SC functioning in MS patients.

To conclude, this study demonstrated that social cognition deficits are present in MS patients since the time of diagnosis, with a persistence over time. These alterations are reflected by early gray matter damage, affecting mainly occipital areas, basal ganglia, and limbic structures. These results highlight the importance of investigating the SC domain in the earliest stages of MS and in the prodromal phases, in addition to traditional neuropsychological assessment batteries.

## Author Contributions

Conceptualization: Stefano Ziccardi. Data curation: Stefano Ziccardi. Formal analysis: Stefano Ziccardi. Investigation: Stefano Ziccardi, Elisa Colato, Maddalena Guandalini, and Agnese Tamanti. Methodology: Stefano Ziccardi, Helen Genova, and Massimiliano Calabrese. Project administration: Stefano Ziccardi. Resources: Helen Genova. Supervision: Helen Genova and Massimiliano Calabrese. Visualization: Stefano Ziccardi and Elisa Colato. Writing – original draft preparation: Stefano Ziccardi. Writing – review and editing: Stefano Ziccardi, Helen Genova, and Elisa Colato.

## Funding Information

This research did not receive any specific funding.

## Conflict of Interest

The authors have no conflict of interest to declare for the present manuscript.

## Data Availability

The data that support the findings of this study are available from the corresponding author upon reasonable request.
